# Regulation of ERBB3/HER3 signaling in cancer

**DOI:** 10.18632/oncotarget.2655

**Published:** 2014-11-02

**Authors:** Kalpana Mujoo, Byung-Kwon Choi, Zhao Huang, Ningyan Zhang, Zhiqiang An

**Affiliations:** ^1^ Texas Therapeutics Institute, Brown Foundation Institute of Molecular Medicine, The University of Texas Health Science Center at Houston, Houston, Texas; ^2^ Current address: Department of Radiation Oncology, Houston Methodist Research Institute, Houston, TX

**Keywords:** HER3, NEDD4, Biomarkers, Monoclonal antibodies, Molecular therapeutics

## Abstract

ERBB3/HER3 is emerging as a molecular target for various cancers. HER3 is overexpressed and activated in a number of cancer types under the conditions of acquired resistance to other HER family therapeutic interventions such as tyrosine kinase inhibitors and antibody therapies. Regulation of the HER3 expression and signaling involves numerous HER3 interacting proteins. These proteins include PI3K, Shc, and E3 ubiquitin ligases NEDD4 and Nrdp1. Furthermore, recent identification of a number of HER3 oncogenic mutations in colon and gastric cancers elucidate the role of HER3 in cancer development. Despite the strong evidence regarding the role of HER3 in cancer, the current understanding of the regulation of HER3 expression and activation requires additional research. Moreover, the lack of biomarkers for HER3-driven cancer poses a big challenge for the clinical development of HER3 targeting antibodies. Therefore, a better understanding of HER3 regulation should improve the strategies to therapeutically target HER3 for cancer therapy.

## INTRODUCTION

### The HER3 receptor

Several excellent reviews describing the role of the epidermal growth factor receptor (EGFR) family members, including HER3, have been published in recent years [1-14]. The focuses of the current review are on the regulation of HER3 expression and function by E3 ubiquitin ligases, and the challenges in developing HER3-targeting antibody cancer therapies due to the lack of biomarkers. EGFR family members play an important role in development and oncogenesis [4, 6, 15]. The HER family is comprised of four closely related transmembrane receptors, EGFR (HER1), HER2 (ERRB2), HER3 (ERBB3) and HER4 (ERBB4) [1]. All receptors except for HER2 interact with multiple ligands. Ligands binding to their receptors trigger a complex and tightly controlled array of signaling pathways involved in the regulation of various cellular functions, including cell proliferation, organ development and organ repair [1, 7]. The HER receptor family is composed of an extracellular domain (responsible for ligand binding), the α-helical transmembrane segment, and the intracellular protein tyrosine kinase domain which mediates interactions with intracellular signaling molecules [8]. In the absence of a ligand, EGFR, HER3 and HER4 exist in a tethered (closed) conformation in which the dimerization domain is not available for the other HER family members [16]. In contrast, HER2 has no known ligand, exists in active extended (open) conformation and can form oligomer, homodimers, and heterodimers with other HER family receptors [8, 9, 15].

Ligands of the HER family receptors are divided into three groups. The first group includes epidermal growth factor (EGF), amphiregulin (AR), and transforming growth factor-α (TGF-α), that bind specifically to EGFR. The second group of ligands include betacellulin (BTC), heparin-binding EGF (HB-EGF), and epiregulin (EPR) which exhibit specificity for both HER1 and HER4 [17]. The third group of ligands include the neuregulins (NRG, also known as Neu differentiation factors, NDFs, or heregulins, HRG) which include two subgroups based on their capacity to bind HER3 and HER4 (NRG1 and NRG2) or only HER4 (NRG3 and NRG4) [18, 19]. NRGs are predominantly found in parenchymal organs and in the embryonic central and peripheral nervous systems. The receptor-ligand binding stimulates the formation of homodimers and heterodimers between the HER receptors, leading to the autophosphorylation of a number of cytoplasmic tyrosine residues. These residues in turn serve as docking sites for many adaptor and signaling proteins for receptor activation [20, 21]. However, HER receptor activation is more complex and exceptions exist. It was reported that EGFR can form homodimers without ligand stimulation, but ligand binding to the homodimers is required for signaling [22]. Ligand independent HER3 signaling has also been reported recently [23].

### Role of HER3 in development

HER family receptors are expressed in cells of epithelial, mesenchymal and neuronal lineages. Additionally, the receptors are expressed in endothelial and cardiac cells, where they play a diverse role in proliferation and differentiation [24, 25]. Furthermore, HER family receptors play a significant role in the development and maintenance of various integrative body systems such as cardiovascular and nervous system as demonstrated by the gene knock out studies. EGFR knock out (KO) mice show embryonic or perinatal lethality. Mice with a naturally occurring germ line mutation in kinase domain of EGFR (known as Waved 2) are viable but display epithelial defects such as wavy hair phenotype. Mutant mice exhibit impaired epithelial development in several organs resulting in peri-implantation death to live progeny with abnormalities in organs such as the liver and the skin depending on genetic background [26, 27]. HER2 KO and HER4 KO animals exhibit embryonic day (ED) 10 lethality due to aberrant cardiac and peripheral nervous system development [28, 29]. Furthermore, HER4 conditional mutant mice display abnormalities in central nervous system and mammary gland [30]. HER2 conditional knockout mice display severely dialated cardiomyopathy with cardiac dysfunction appearing by second post natal month [31]. HER3 KO mice exhibit ED13.5 lethality due to defective valve formation, pronounced heart defects (double-outlet right ventricle and atrio-ventricular cushion defects) and vasculature abnormalities [32]. Specifically, HER3 knock out mice have hypoplastic cardiac cushions with decreased mesenchyme [33]. HER3 is expressed on endocardial cushion cells and mesenchymal cells undergoing EMT. This is in contrast to EGFR, HER2 and HER4 expression which is largely limited to cardiomycytes during the critical cushion forming period [25].

NRGs and HER receptors are implicated in control of the growth and development of Schwann cells (cells which wrap around the axons to provide electrical insulation). Further, homozygous HER3 mutant embryos lack Schwann-cell precursors, which leads to the cell death of motor and sensory neurons at the later stages of development in HER3 mutant pups. This abnormality thereby implicates an important role of HER3 in nervous system development [34]. Furthermore, the role of HER3 in mouse mammary gland development was investigated by transplanting mammary buds from HER3 ^−/−^ embryos into cleared mammary fat pads of immune compromised wild type mice. HER3 ^−/−^ buds only partially filled the mammary fat pad, however, lobuloalveolar development of HER3 ^−/−^ transplanted glands was normal [35]. The broader conclusion of the study was that mammary outgrowth defect in HER3 ^−/−^ was associated with a decrease in the size of the terminal end buds, increase in the branch density and increase in the number of terminal buds. In addition, although a lack of HER3 did not affect the proliferation rate, it did increase the apoptosis in HER3 ^−/−^ terminal end buds, suggesting HER3 is required for ductal morphogenesis in the mouse mammary gland [35]. The phenotype induced by a lack of HER3 in the development of mouse mammary gland was more pronounced than the knock out of any of the other HER receptors, alluding to the significance of HER3 in mammary development [35]. This conclusion was supported by the work by other investigators. In another study, when HER3 expression in the mammary gland was inhibited by Cre recombinase, the mammary glands analyzed after 4 and 8 weeks also exhibited lower ductal density, fewer branches and fewer terminal bud end buds [36]. An additional study demonstrated HER3 maintains the balance between luminal and basal breast epithelium. A loss of HER3 in the mouse luminal mammary epithelial cells lead to impaired AKT and MAPK signaling as well as reduced luminal proliferation and survival [37].

### HER3 overexpression, activation, and mutations in cancer

HER3 plays an important role in cell proliferation and survival [5]. HER3 was identified based on its homology to EGFR [38, 39] and thereafter it was determined that HER3 has an impaired kinase function [40]. Neuregulin binding (NRG) to HER3 induces heterodimerization of HER3 with other EGFR family receptors, particularly HER2, resulting in the phosphorylation of tyrosine residues of the C-terminal tail of HER3 [41]. Phosphotyrosines of HER3 provide binding sites for PI3K, Shc and other HER3 interacting proteins [42, 43], which mediate activation of the PI3K/AKT and Ras/Raf/MAPK pathways [44, 45]. Although HER3 has been suggested to play a role in oncogenesis for many years [46], its absolute significance in cancer biology has begun to emerge in recent years [1, 10]. HER3 was initially studied in HER2 - amplified breast cancers [46, 47]. HER3 was also detected in various cancers under the conditions of acquired resistance to other HER family member therapeutic interventions. For example, a study established that resistance to the EGFR kinase inhibitor gefitinib in lung cancer leads to amplification of the MET proto-oncogene. This augmentation of the MET proto-oncogene was due to the activation of HER3 phosphorylation and PI3K activation in an EGFR and HER2 independent manner [48].

Overexpression of HER3 has been reported in primary cancers and in cultured cells including the carcinomas of breast, ovarian, prostate, colon, pancreas, stomach, oral cavity and lung [11]. Studies have shown that 50-70% of human breast cancers have detectable HER3 levels as evaluated by IHC and activated HER3 is usually co-overexpressed with HER2 in breast cancers [49-51]. Oncogenic mutations in HER3 gene were reported in human colon and gastric cancers [52, 53] and some of these mutations were shown to be gain of function mutations. The study further provided evidence of oncogenic activity with HER3 for Q809R in gastric cancer [52]. Furthermore, although HER3 mutation at V714M was identified in non-small cell lung cancer (NSCLC) patients and S846I mutation was identified in colon cancer, oncogenic function of these mutants has not been tested [52].

Increased levels of HER3 mRNA in colon cancer cell lines and colorectal tumors have been reported [54] and HER3 expression can be detected in 30-90% of colorectal tumors [55, 56] where HER3 is frequently co-expressed with HER2 and EGFR [57]. Moreover, signaling through EGFR and HER3 is thought to play a role in colon cancer [2] and HER3 expression has been shown to correlate disease progression in colon cancer patients [58]. Furthermore, HER3 down regulation by RNA interference and anti-HER3 antibody treatment led to inhibition of cell proliferation, migration, and invasion, G2-M cell-cycle arrest and induction of apoptosis in colon cancer cell lines [58]. A recent study clearly demonstrated that HER3 knockdown induces cell cycle arrest and apoptosis of colon cancer cell lines by activation of Bak and Bax [59]. These studies collectively allude to the critical involvement of HER3 in colon cancer progression [58].

Similarly, other studies report that HER3 mRNA is increased in some ovarian cancer [60] patients and 16% of ovarian tumors show overexpression of HER3 protein compared to normal ovarian samples [61]. Furthermore, autocrine regulation of cell growth has been shown by overexpression of HER3 ligand neuregulin (NRG) [62]. Direct correlation between HER3 overexpression and poor overall prognosis of ovarian cancer has also been reported [63].

Additional studies show HER3 overexpression (as evaluated by IHC) is associated with poor prognosis of lung adenocarcinoma [64] and with decreased survival of patients with stage I-IIIA of NSCLC [65]. Constitutive activation of HER3 is also observed in a number of lung adenocarcinoma cell lines that co-express HER2 [66]. HER3 transgenic mice also reveal the significance of HER3 overexpression in lung tumorigenesis [67]. Furthermore, double transgenic mice with human HER3 and rat HER2 exhibit spontaneous lung tumors similar to single transgenic mice, albeit a shorter latency period thereby further demonstrates the involvement of HER2/HER3 dimers in the initiation of lung carcinogenesis [67]. Among other cancers, high HER3 expression has been reported in melanoma metastases using IHC staining [68] and HER3 has been shown to be frequently expressed in human melanoma cells [69]. Furthermore, using gene expression microarray analysis, HER3 was one of the small group of genes up regulated in melanoma [68].

Similarly, a study using microarray analysis has shown increased expression of HER3 in prostate cancer compared to a normal prostate [70] and further studies using IHC reveal that 90% of human prostate cancer show significant HER3 staining [71-73] therefore pointing to the importance of HER3 signaling in prostate cancer.

A meta-analysis study linking HER3 overexpression and survival in solid tumors (using the data from immunohistochemistry) revealed the median percentage of cancers with HER3 overexpression was 42.2% and HER3 was associated with worse overall survival (OS) at both the 3 year and the 5 year period [74]. These studies collectively show involvement of HER3 in progression of various cancers.

### HER3 and resistance to cancer therapies

As mentioned earlier, HER3 activation is associated with resistance to HER2 targeting tyrosine kinase inhibitors in breast cancer [75, 76]. It has been shown that HER3 and PI3K/AKT signaling escape the inhibition exerted by tyrosine kinase inhibitors of the HER family members. This is partly thought to be attributed to a compensatory shift in the HER3 phosphorylation-dephosphorylation equilibrium, driven by AKT mediated negative feedback signaling [75]. Similarly, hepatoma cells are able to overcome IGF1R inhibition through HER3 activation in an EGFR-dependent mechanism, suggesting HER3 is an important mediator in acquired resistance to anti-IGF1R therapy [77]. Studies have also shown that HER2/HER3 heterodimers lead to aberrant activation of androgen receptor, contributing to the development of hormone resistant prostate cancer [78]. Furthermore, EBP1, a HER3 binding protein acting as co-repressor of androgen receptor (AR), has shown to be decreased in hormone refractory prostate cancer [78]. Recent studies have also shown that androgen-independent prostate cancer cells modulate the expression of HER receptors and ligands. This regulation of HER receptors sustains the growth of cancer cells after the EGFR blockade, and HER3 plays a central role in mediating such resistance to EGFR inhibition [79, 80]. A recent study has shown neratinib (pan HER inhibitor) is able to overcome trastuzumab resistance in HER2 amplified breast cancer. Furthermore, although neratinib decreased the activation of all four HER receptors initially, HER3 and AKT were reactivated in 24 hours. However, HER3 and AKT were inhibited by the combination of trastuzumab and neratinib [81]. Another study using antibodies against HER3 and EGFR TKIs show the synergistic effect on cell proliferation *in vitro* and the inhibition of tumor growth in mouse xenograft models of non-small cell lung cancer [82]. This synergistic effect suggests the combination treatment of HER3 antibodies and EGFR TKIs is a promising approach to pursue in the clinic.

### Regulation of HER3

**Figure 1:**
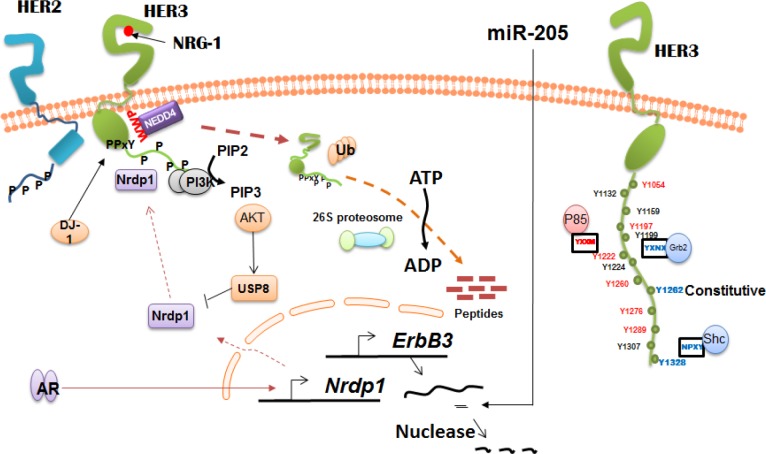
Regulation of HER3 expression and function HER3 is regulated by a number of E3 ubiquitin ligases such as NEDD4 and Nrdp1 by mediating its ubiquitination and degradation. AR negatively regulates HER3 levels by modulating Nrdp1 levels in androgen dependent prostate cancer. HER3 is also under the regulation of number of micro RNAs including miR-205.

The regulation of HER3 at various levels is depicted in Figure 1. The protein expression of HER3 is modulated at transcriptional, post transcriptional and post translational levels [3]. Regulation of HER3 expression and signaling using HER3 interacting proteins such as E3 ubiquitin ligase NEDD4, Nrdp1 and Nrdp1 regulator USP 8 [83] has emerged from our recent studies [84] and reports from other investigators [10, 83, 85, 86]. Additionally, we [84] and others have used therapeutic HER3 antibodies as probes to study the implication of HER3 inhibition/down-regulation in preclinical models of human cancers [2, 13, 87]. Our laboratory has demonstrated intracellular domains/C-terminal tail of HER3 plays a key role in dimerization of HER2/HER3 and in the activation of downstream signaling pathways. This was achieved by construction of HER3/HER2 chimeric receptors which were engineered by replacing the HER3 kinase domain (HER3-2-3) or by replacing both kinase domain and C-terminal tail (HER3-2-2) with the HER2 counterparts. Our results suggest intracellular domains play a crucial role in establishing the function of HER3 as an allosteric activator and its role in downstream signaling [88]. We further reported an HER2 antibody which blocks HER2/HER3 dimerization can induce ligand independent HER3 dimerization with EGFR in both low and high HER2 expressing cancer cells. Furthermore, our results suggest HER3 plays an important role in sensing the perturbation of HER2 signaling caused by HER2 antibodies and in maintaining equilibrium of EGFR family mediated signaling [80].

When phosphorylated, the 14-tyrosine residues present on the C-terminal tail of HER3 are potentially capable of docking numerous SH2 or PTB binding proteins involved in a number of signaling pathways [3, 89, 90]. One of the most critically important signaling activity of HER3 is its unique ability to activate PI3K/AKT pathway by six consensus phospho tyrosine sites present on the C-terminal tail that bind to the SH2 domain of the regulatory subunits of PI3K [42, 43]. A previous study reported the generation of several HER3 deletion and Tyr-Phe mutations, and observed that a single YXXM motif was necessary and sufficient for the association of HER3 with p85 [44]. Another study demonstrating the role of HER3 in the early stages of breast epithelial transformation showed the loss of HER3 (Cre mediated HER3 ablation) prevented the progressive transformation of HER2, overexpressing mammary epithelium [91]. Further, the loss of HER3 impaired AKT and ERK phosphorylation in pre-neoplastic HER2, overexpressing mammary glands. The tumors which were rescued by re-expression of HER3 were only partially blocked by an HER3 mutant (6 tyrosine to phenyalanine mutations), blocking the interaction of HER3 to PI3K [91]. Another study exploring the significance of HER3/PI3K in mammary development generated a mouse model carrying a mutant HER3 allele lacking 7 known PI3K binding sites (ErbB3^Δp85^). Homozygous mice (ErbB3^Δp85^) of this study further exhibited an early growth defect and impairment of mammary epithelial outgrowth [92]. However, all the female mutant mice developed metastatic HER2 induced mammary tumors, thereby suggesting although HER3 associated PI3K activity is critical for mammary development, it is not required for HER2 induced mammary tumor progression [92]

Additional studies have shown HER3 is under the regulation of several micro RNAs (miRNA) including miR205, miR125a and miR125b [93, 94]. Micro RNAs are known to regulate gene expression of many proteins in cancer by either functioning as an oncogene or a tumor suppressor gene. A study has shown miR205 directly targets the HER3 receptor and inhibits AKT activation. The same study showed the reintroduction of miR205 in breast cancer cells was able to increase the TK inhibitors responsiveness [93]. A recent study using bioinformatics analysis showed miR205 binds to the 3′- untranslated regions of human HER3 mRNA. Additionally, the regulation of miR205 led to the reduction of HER3 protein expression [95]. Similarly, overexpression of miR125a and miR125b also suppressed HER2 and HER3 at both mRNA and protein levels. Enforced expression of miR125a and miR125b caused phenotypic changes (inhibition of anchorage dependent growth, proliferation, migration and invasion) in the tested breast cancer cells [94].

Furthermore, a recent study demonstrated hyperglycemia leads to epigenetic up-regulation of the *NRG1* gene (HER3 ligand). NRG1-HER3 autocrine loop activates HER3 signaling pathways, leading to more malignant progression of tumor cells under hyperglycemia (and even after a return to euglycemic conditions). These results therefore suggested NRG-HER3 axis mediates hyperglycemic memory effects in breast cancer [96].

### Regulation of EGFR family members by ubiquitin-proteasome pathway

The ubiquitin-proteasome pathway regulates both normal and pathological cellular processes such as proliferation, differentiation, cell cycle and apoptosis [97]. Numerous studies have shown that the ubiquitin-proteasome pathway plays a significant role in cancer initiation and progression by regulating protein levels and activities of both tumor promoting and suppressing factors [98-104]. E3 ubiquitin ligases are known to regulate EGFR family receptors [10]. For instance, the E3 ligase Cbl associates with EGFR and, upon activation with EGF and the interaction with Cbl, EGFR undergoes lysosomal degradation [105]. Similarly, CHIP E3 ligase associates with HER2 to promote its ubiquitination and degradation [106]. Previous studies have reported that an RING finger E3 ubiquitin ligase Nrdp1 interacts with HER3 and promotes HER3 ubiquitination and degradation via proteasome in breast and prostate cancer cells [85, 107, 108]. Moreover, both WWP1 and ITCH E3 ligases were shown to be involved in ubiquitination and degradation of HER4 [109, 110]. Further, a recent study demonstrated the enhancement of the chemotherapeutic response in non-small cell lung cancer models by blocking NRG and other ligand mediated HER4 signaling. This augmentation implies the role of ligand-dependent HER4 signaling in disease relapse [111]. Our recent study identified E3 ligase NEDD4 (neural precursor cell expressed developmentally down-regulated 4) as a novel interaction partner of HER3 [84].

### Role of NEDD4 and Nrdp1 in HER3 regulation

E3 ligase NEDD4 is a member of ubiquitin-proteasome E3 ligase of the HECT family (homologous to E6-AP COOH terminus). Similar to other reported members in the family, NEDD4 contains a C2 domain (N-terminal), 4 WW domains and a C-terminal HECT domain [112-114]. The C2 domain of NEDD4 is a Ca^2+^-dependent phospholipid binding domain [115], and it is also involved in protein localization and trafficking [114]. The WW domains are involved in the substrate recognition and specifically interact with the poly-proline type II helix formed by the PPXY motif of the substrate [116-118]. The tyrosine (Y) in PPXY motif has been shown to form hydrogen bonds with the WW domains, contributing to the interaction between the substrate and NEDD4 [119]. A recent study showed that NEDD4 mediated ubiquitination and degradation of activated tyrosine kinase receptor FGFR1, thereby regulating its endocytosis and signaling [120]. Further, NEDD4 acts as an E3 ligase for epithelial sodium channels to regulate its ubiquitination and degradation [113, 121]. Another study reported NEDD4 as an E3 ligase for EGFR family member HER4 in Madin-Darby canine kidney II cells [122].

**Figure 2:**
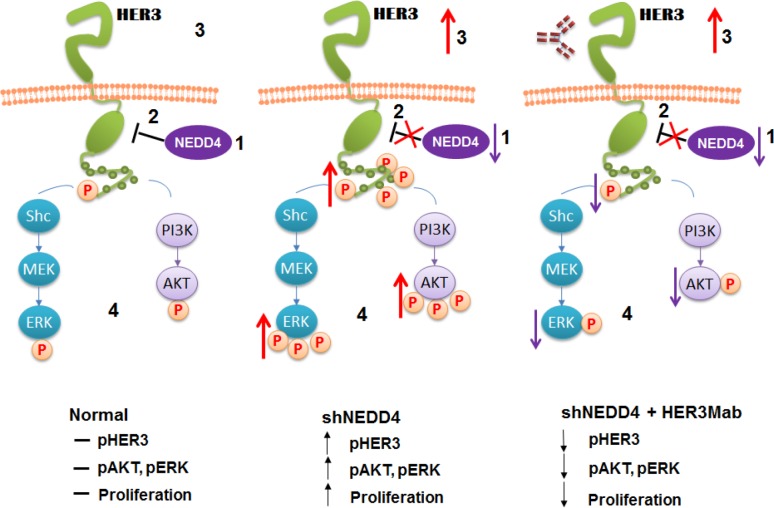
Working model for the HER3/NEDD4 interaction (adapted from Zhao et al., Oncogene, 2014) Physiological levels of NEDD4 negatively regulate the steady-state levels of HER3 resulting in normal HER3, AKT1 and ERK1/2 phosphorylation upon NRG-1 activation. Upon shNEDD4 knockdown increased HER3 levels and enhanced NRG-1 dependent HER3, AKT1 and ERK1/2 phosphorylation is observed. The increased HER3, AKT1 and ERK1/2 phosphorylation induced by NEDD4 knockdown can be neutralized by an anti-HER3Mab.

Our recent studies demonstrated NEDD4 is a novel interaction partner of HER3 and functions as an E3 ubiquitin ligase for regulation of the steady state levels of HER3 [84]. Cancer cells with NEDD4 knockdown (shRNA) exhibited increased HER3 levels that promoted HER3 mediated signaling, cell proliferation and migration as depicted in Figure 2. C-terminal tail of HER3 interacted with the WW domains of NEDD4 in a neuregulin-1 independent manner. shRNA knockdown of NEDD4 elevated HER3 levels, resulting in increased HER3 signaling and cancer cell proliferation *in vitro* and *in vivo* in a MCF-7 mouse xenograft model. Further, an inverse relationship between the HER3 and NEDD4 levels was observed in ductal cells of prostate cancer tumor tissues. In addition, up-regulated HER3 expression by NEDD4 knockdown sensitized cancer cells for growth inhibition by an anti-HER3 antibody. This sensitization suggested low NEDD4 levels may predict activation of HER3 signaling and therefore effectiveness of HER3 antibody therapies [84]. Lack of biomarkers is a major obstacle in early detection and treatment of various malignancies. Therefore, biomarker validation for patient stratification of molecular targeted therapies is of utmost clinical importance and we speculate that NEDD4 may be a responding marker for HER3 therapies.

Nrdp1 is a 317 amino acid protein that contains “tripartite” motif consisting of an N-terminal RING domain, two zinc finger domains (B-Box) and a coiled-coil segment [123]. Functionally, this class of E3 ligases is involved in a number of cellular processes including apoptosis, cell cycle regulation and transcriptional control [124]. Nrdp1 has been shown to differentially ubiquitinate various substrates including MyD88, Parkin, BRUCE, HER3/ErbB3 and others [125] [126].

Previous studies show Nrdp1 also functions as an E3 ubiquitin ligase to regulate steady-state ErbB3 levels by mediating its ligand-independent ubiquitination and degradation. This was demonstrated by studies where the overexpression of wild-type Nrdp1 suppressed cellular ErbB3 levels, however, dominant negative Nrdp1 form potently augmented the receptor levels [107, 108]. These studies suggest that Nrdp1 constitutively internalizes ErbB3 receptors to divert them from recycling to the lysosomal degradation pathway [127]. Nrdp1 has been shown to mediate ubiquitination of neuregulin induced HER3 in breast cancer cells [83]. Additional study has revealed overexpression of Nrdp1 in breast cancer cells suppresses HER3 levels, cell proliferation, motility and inhibition of signaling pathway [128]. Moreover, Nrdp1 protein is suppressed in approximately 60% of the tumors compared to the patient matched normal tissues, therefore showing a strong inverse correlation between Nrdp1 and HER3 [128]. Further studies in prostate cancer cells show androgen receptor (AR) regulates HER3 levels by promoting its degradation by regulating Nrdp1 transcription. Interestingly, AR regulates Nrdp1 levels transcriptionally in androgen-dependent but not in castration resistant prostate cancer [85].

Furthermore, in addition to the lysosomal degradation pathway, involving degradation of a mature HER3 receptor, Nrdp1 preferentially associates with nascent HER3 to mediate its degradation through endoplasmic reticulum associated degradation pathway (ERDP). This phenomena was shown by the studies where both Nrdp1 and HER3 proteins co-localized at the endoplasmic reticulum [129]. ATPase VCP/p95 of the ERDP was an essential component of the Nrdp1 mediated HER3 degradation. This was represented by studies where functional disruption of VCP led to Nrdp1 dependent accumulation of ubiquitinated HER3 but blocked receptor degradation. Collectively, these studies have proposed a novel mechanism where Nrdp1by an ER-localized degradation pathway regulates signaling-competent HER3 [129]. The authors have further proposed a model where Nrdp1 could perform as a committed regulated component into ERAD machinery to degrade HER3 when signaling by receptor might be counterproductive.

Our recent study indicated NEDD4 knockdown [84] of breast and prostate cancer cells did not impact Nrdp1 expression, which prompted us to speculate the two ligases may interact with HER3 at different sites. Our results further show NEDD4 interacts with the C-terminal tail of HER3. In contrast, a previous study [130] has shown Nrdp1 binds with HER3 either on the juxta-membrane domain or kinase domain. Thus, our research suggests that Nrdp1 and NEDD4 could regulate HER3 levels by different mechanisms.

### Targeting HER3 for cancer therapy – preclinical models and clinical studies using antagonist HER3 antibodies

**Figure 3:**
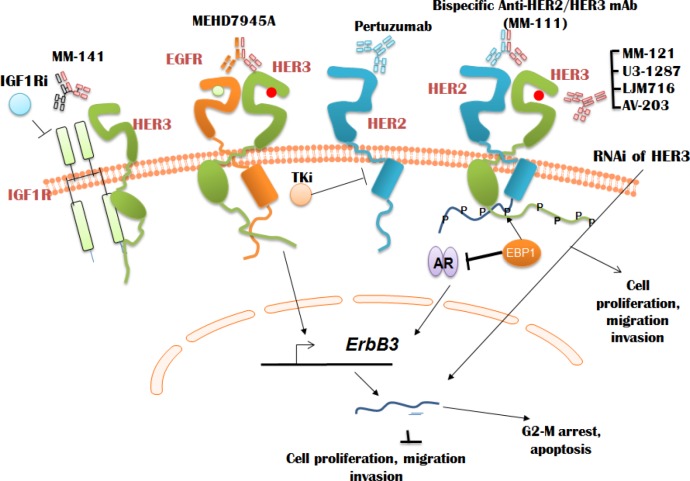
Regulation of HER3 expression and signaling using antibodies and tyrosine kinase inhibitors Various monoclonal antibodies against HER3 which bind at various domains of HER3 (described in the text) to block HER3 function, cancer cell proliferation, migration and invasion have been described. Some of HER3 monoclonal antibodies such as MM121 in clinical trials demonstrated that targeting neuregulin-positive tumors with MM-121 sensitizes patients to exemestane, erlotinib and paclitaxel in metastatic breast, lung and ovarian cancers, respectively, and significantly lowers their risk of tumor progression.

As described below and shown in Figure 3, a number of HER3 antagonist antibodies have been evaluated in preclinical tumor models. More recently, some of antibodies (most notably MM121) have also been investigated in clinic against various types of cancer. Studies have shown that monoclonal HER3 antibody MM121 which targets ligand-dependent HER3 activation [131] exhibits substantial tumor growth arrest against various xenograft tumors including prostate (DU-145), ovarian (OVCAR-8) and renal (ACHN) cancer [131-133], albeit without inducing complete tumor regression. The cause for lack of tumor regression by MM121 could be attributable to the lack of dependence on HER3 or the limited potency of MM121 towards activated HER3 [13]. Further, the importance of HER3/NRG autocrine loop was established by demonstrating that both MM121 and HER3 targeted RNAi approaches slowed growth of ovarian carcinoma (OVCAR8) cells *in vitro* and *in vivo*. Although RNAi-targeted HER3 cells were able to cause complete tumor regression compared to MM121 treatment alone, suggesting single antibody treatment may not be sufficient for tumor regression [133]. Recently presented clinical trials with MM121 and tyrosine kinase inhibitors and chemotherapeutic drugs show the mRNA expression of neuregulin (NRG; HER3 ligand which binds to and activates HER3) [134] is associated with poor response to platinum-resistant ovarian cancer, ER/PR^+^ HER2^−^ breast cancer and EGFR wild-type non-small cell lung cancer [135]. Furthermore, drug resistance to multiple standard care therapies induced by neuregulin were active in ~30-50 percent of patients tested, suggesting neuregulin-driven HER3 signaling plays a significant role in promoting resistance to multiple therapeutic agents. These studies further demonstrate targeting neuregulin-positive tumors with MM-121 sensitizes patients to exemestane, erlotinib and paclitaxel in metastatic breast, lung and ovarian cancers, respectively, and significantly lowers the risk of tumor progression. These clinical studies therefore identified neuregulin as a patient response biomarker for MM-121. Furthermore, the clinical trials concluded that patients with low HER2 exhibited the maximum benefit from MM-121 (NCT00994123), (NCT01447225).

Another monoclonal antibody, LJM716, is selective for an epitope on HER3 ECD (domains II and IV). This antibody can lock HER3 in an inactive conformation, thereby preventing both ligand-dependent and -independent activation of HER3 [136]. Furthermore, LJM716 antibody treatment resulted in significant growth inhibition in various xenograft models of ligand-dependent and ligand-independent modes of HER3 activation, with more than 80% growth inhibition in the HER2^+^ BT474 xenograft [136]. Other ligand driven models such as FaDu also showed significant *in vivo* growth inhibition with LJM716 [136]. This antibody has recently completed Phase I clinical trial in patients with squamous cell carcinoma of head and neck or HER2^+^ breast cancer or gastric cancer (NCT01598077).

AMG-888 (U3-1287), which targets ligand-induced phosphorylation of HER3, has also been investigated for its anti-tumor activity against various models of breast cancer and NSCLC [2, 137]. Preclinical studies with U3-1287 demonstrate tumor inhibition in A549 model and partial inhibition in FaDu model, suggesting the antibody might be more effective against HER2+/EGFR+ amplified models relative to the NRG driven models. Study of U3-1287 with erlotinib in patients with advanced stage NSCLC has been completed with no study results currently posted (NCT01211483). Further, there is an ongoing Phase II clinical trial using U3-1287, trastuzumab and paclitaxel in HE2^+^ breast cancer (NCT01512199).

AV-203 is a humanized IgG1 antibody that inhibits ligand-dependent HER3 activation and its downstream molecule AKT. AV-203 also shows potent tumor growth inhibition in a number of xenograft models where HER3 is activated by ligand NRG1 or HER2 overexpression [138]. This antibody is also currently in clinical trials for advanced solid tumors (NCT01603979). Furthermore, TK-A3 and TK-A4 (anti HER3 humanized antibodies) have been investigated for their anti-tumor activity against BxPC3 (pancreatic) and some genetically engineered (GEM) models [87].

MM-111 is a bispecific antibody (targets both HER2 and HER3) (developed by using computational modeling) that forms trimeric complex with HER2 and HER3 [139]. Furthermore, this antibody was shown to potently and specifically inhibit HER3 signaling in HER2 positive tumors. MM-111 exhibited antitumor activity in preclinical models dependent on HER2 overexpression and the antibody was shown to exert increased antitumor activity when combined with HER2 antibody trastuzumab or tyrosine kinase inhibitor (TKI) lapatinib targeting EGFR and HER2. Currently, a Phase I clinical trial of MM-111 in combination with trastuzumab is being conducted in patients with advanced, refractory HER2 amplified and neuregulin positive breast cancer (NCT01097460). In addition, another Phase I study of MM-111 in combination with multiple treatment regimens in patients with advanced HER2 positive solid tumors is also being conducted (NCT01304784).

Similarly, a dual action monoclonal antibody MEHD7945A targeting EGFR and HER3 was examined in a number of xenograft models. Among the responders were pancreatic (BxPC3) and NSCLC (NCI-H292 and Calu-3) cell lines [140]. Furthermore, a recent study showed that by antagonizing EGFR and HER3 using MEHD7945A, an enhanced response to PI3K inhibitor (GDC-0941) and AKT inhibitor (GDC-0068) in triple negative breast cancer was observed. This response therefore emphasized the concomitant blockade of EGFR, PI3K and AKT pathway should be investigated in clinic [141]. This antibody has also entered Phase I & II trials for head and neck and metastatic epithelial cancers (NCT01577173).

Previous studies indicated an aberrant activation of IGFR in many cancers associated with HER targeted therapies [77, 142]. These studies prompted the generation of bispecific antibody MM-141 against HER3 and IGFR. MM-141 has been shown to block binding of NRG to HER3 and IGF1/2 binding to IGFR, causing inhibition of PI3K/AKT/mTOR pro-survival signaling in preclinical cancer models [143]. Furthermore, MM-141 has been proven to inhibit pancreatic tumor cell growth and potentiate of effect of gemcitabine in various preclinical models [144]. In addition, MM-141 has exerted its anti-proliferative activity against melanoma [145], and this antibody has also attenuated tumor growth and potentiated the activity of mTOR inhibitor everolimus in mouse models of anti-hormone therapy-resistant ER/PR^+^ breast cancer [146]. Furthermore, previous studies with MM-141 have shown the ablation of HER3 signaling results in the inhibition of PI3K/AKT dependent mammary carcinogenesis and ERK1/2 phosphorylation in pre-neoplastic HER2 overexpressing mammary glands and tumors [91, 147]. Patients are being currently recruited for a Phase I clinical trial of MM-141 for advanced solid tumors (NCT01598077).

### SUMMARY

A recent meta-analysis study has revealed the median percentage of cancers with HER3 overexpression is over 40%. Furthermore, HER3 was found to be associated with a lower overall survival rate after 3-5 year period of initial diagnosis. Regulation of HER3 expression and signaling by HER3 interacting proteins such as E3 ubiquitin ligase NEDD4, Nrdp1 and Nrdp1 regulator USP-8 has emerged from our studies and from the studies of other investigators. These studies suggest that both NEDD4 and Nrdp1 regulate steady state levels of HER3 by proteasomal degradation. Furthermore, we and others have used therapeutic HER3 antibodies as probes to study the implication of HER3 inhibition/down-regulation using NEDD4 and Nrdp1 KD preclinical models of various human cancers. Recent clinical studies reveal that targeting NRG-positive tumors with monoclonal antibody MM-121 sensitized patients to TKI and chemotherapeutic drugs and significantly lowered their risk of tumor progression. These clinical studies therefore identified neuregulin as a patient response biomarker for MM-121. Taken together, although there are several promising HER3 antibodies, greater preclinical and possibly clinical benefits may be attained by combining the HER3 antibodies with other antibody and/or small molecule TKI inhibitors. Bi-specific antibodies such as MM-111, MEHD7945A, and MM-141 are also promising because of their capability to simultaneously target HER3 and other partners of the tyrosine kinase receptors HER family such as HER2, EGFR, and IGF1R.
